# Cognitive behaviour therapy (CBT) for anxiety and depression in adults with mild intellectual disabilities (ID): a pilot randomised controlled trial

**DOI:** 10.1186/1745-6215-12-95

**Published:** 2011-04-14

**Authors:** Angela Hassiotis, Marc Serfaty, Kiran Azam, Andre Strydom, Sue Martin, Charles Parkes, Robert Blizard, Michael King

**Affiliations:** 1 Department of Mental Health Sciences, University College Medical School, Charles Bell House, 67-73 Riding House Street, London W1W 7EY, UK; 2 Department of Mental Health Sciences, University College Medical School, Royal Free Hospital, Pond Street, London NW3 2Q, UK; 3 Camden Learning Disabilities Service, Bedford House, 125 Camden High Street, London, NW1 7JR, UK; 4 Islington Learning Disabilities Partnership, 52d Drayton Park, Islington, N5 1NS, UK

## Abstract

**Background:**

Several studies have showed that people with intellectual disabilities (ID) have suitable skills to undergo cognitive behavioural therapy (CBT). Case studies have reported successful use of cognitive behavioural therapy techniques (with adaptations) in people with ID. Modified cognitive behavioural therapy may be a feasible and effective approach for the treatment of depression, anxiety, and other mood disorders in ID. To date, two studies have reported group-based manaulised cognitive behavioural treatment programs for depression in people with mild ID. However, there is no individual manualised programme for anxiety or depression in people with intellectual disabilities. The aims of the study are to determine the feasibility of conducting a randomised controlled trial for CBT in people with ID. The data will inform the power calculation and other aspects of carrying out a definitive randomised controlled trial.

**Methods:**

Thirty participants with mild ID will be allocated randomly to either CBT or treatment as usual (TAU). The CBT group will receive up to 20 hourly individual CBT over a period of 4 months. TAU is the standard treatment which is available to any adult with an intellectual disability who is referred to the intellectual disability service (including care management, community support, medical, nursing or social support). Beck Youth Inventories (Beck Anxiety Inventory & Beck Depression Inventory) will be administered at baseline; end of treatment (4 months) and at six months to evaluate the changes in depression and anxiety. Client satisfaction, quality of life and the health economics will be secondary outcomes.

**Discussion:**

The broad outcome of the study will be to produce clear guidance for therapists to apply an established psychological intervention and identify how and whether it works with people with intellectual disabilities.

**Trial registration:**

ISRCTN: ISRCTN38099525

## Background

The level of psychopathology in people with intellectual disabilities (ID) is higher than in the general population [1] with depression, anxiety and mixed affective disorder being the commonest diagnoses. The prevalence rates for affective disorder and anxiety in a random community sample of 90 adults with ID using a structured assessment and International Classification of Diseases (ICD-10) criteria were 8.8% and 14.4% respectively [2]. Two recent studies which compared birth cohorts of adults with and without ID found a four [3] to six fold [4] increase in common affective disorders in adults with mild ID.

It has been suggested that psychotherapy in general is not effective in people with ID [5-9]. This is possibly due to long-held assumptions that people with ID have insufficient intellectual capacity to use talking therapies as well as a lack of instruments to measure change in symptoms, "therapeutic disdain" [10], a reluctance to work collaboratively and to understand the mental world of the patient [11]. However, in recent years, there has been a greater focus on the psychological needs of people with ID, and therapeutic optimism borne out of greater understanding of the adaptations required to improve interventions for this group. Recent limited evidence suggests that at least a year's psychodynamic orientated therapy with adults across the range of intellectual disability leads to positive outcomes in improving emotional intelligence [12].

Evidence from work with children suggests that a mature cognitive function may not be necessary for an individual to make use of cognitive behaviour therapy (CBT) [13]. Indeed some research has found that people with mild to moderate intellectual disability are able to establish links between thoughts and feelings, an important rationale underpinning CBT [14].

Successful use of CBT has been reported in case studies of adults with mild intellectual disability who offended exhibited problem behaviours [15], anxiety [16] or suffered with long term psychotic symptoms [17]. A meta-analysis of the efficacy of psychotherapy in adults with intellectual disability [18] found that cognitive behavioural methods were used in 13% of all the studies reviewed. The outcome of this meta-analysis suggested an effect size for CBT of 3.08. However most CBT interventions have focused on the behavioural rather than cognitive aspects of the interventions [19,20] looking at observable behaviour rather than psychological change as the outcome measure.

There are many challenges to conducting well designed randomised trials (RCTs) in this field, including ethical objections, the need to involve carers and lack of resources for the intervention [21]. Only two RCTs have been published using CBT in ID and these addressed anger management in secure units [22] and in a community setting [15] using self management and cognitive techniques [23-25].

Cognitive behaviour techniques in people with ID are often adapted to include use of pictures, role plays and simpler questioning styles. Therapy may need to proceed at a slower pace with therapy sessions shorter than the usual 50 minutes [26]. We are not aware of any general treatment manuals for CBT in adults with ID despite increasing demand for training.

In summary, the evidence for efficacy of CBT in people with mild ID is weak and based on small case studies or clinical trials that have significant methodological shortcomings [27]. In addition, positive outcome maybe associated with spontaneous remission in symptoms of depression and/or anxiety [28,28]. Reliable and valid outcome measures are rarely used [18], and the treatments are not standardised (see Journal of Applied Research in Intellectual Disabilities special issue, 2006).

The rationales for proposing this study are:

• A poor evidence base for efficacy of psychological treatments in people with ID;

• Exclusion from access to established health interventions for common mental disorders of this population [30];

• Little understanding of how and why treatments established in other populations might be effective in adults with ID.

This is a preliminary study in order to adapt and operationalise a standard intervention for a specific service user group. The broad outcome of the study will be to produce clear guidance for CBT trained therapists to apply the intervention and identify how and whether it works in this group.

### Hypothesis

Cognitive behaviour treatment is more clinically and cost effective than treatment as usual for depression, anxiety and mixed affective states in adults with mild intellectual disabilities.

### Objectives

The aims of the present trial are:

• To develop a manual outlining how to carry out cognitive behavioural treatment for common mental disorders in adults with mild ID.

• To determine the feasibility of conducting a randomised controlled trial of individual CBT versus treatment as usual (TAU) for the treatment of depression and anxiety in adults with mild ID.

• To use data arising on differences between CBT and TAU to inform a power calculation for numbers in a definitive randomised controlled trial.

• To identify the clinically useful elements in the treatment manual.

• To investigate satisfaction with the intervention and service costs.

## Methods/Design

### Study Design

**Figure 1:**
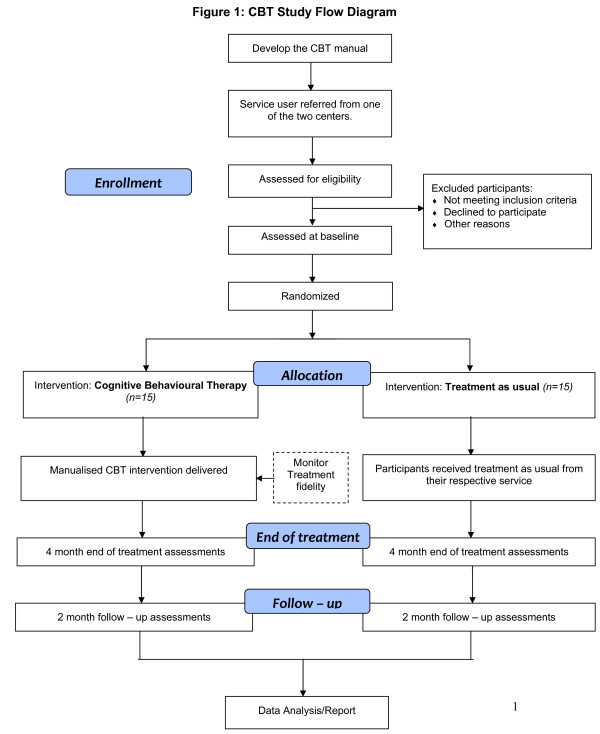
**CBT Study Flow Diagram**.

Potential participants' referrals will be taken from professionals across two local intellectual disability services in the London boroughs of Camden and Islington in the United Kingdom, as well as new referrals that fulfill inclusion criteria. Participants will be randomly allocated to Cognitive Behavioural Therapy plus treatment as usual (CBT) or Treatment as Usual (TAU). Figure 1 shows the trial design.

### Screening and participant recruitment

A total of thirty (*n *= 30) participants will be recruited for the study. Potential participants will be screened for the trial using the depression and anxiety components of the Mini Psychiatric Assessment Schedules for Adults with Developmental Disabilities (Mini PAS-ADD) [31]. Those with scores of more than 10 for depression and more than 7 for anxiety will be eligible and randomized into the study. The range of scores indicating severity is between 11 and 32 for depression and 7-18 for anxiety.

Inclusion criteria:

• Participants will be adults (over 18 years of age) who have mild intellectual disability (as determined on the service register).

• Have a disorder according to one of the following International Classification of Diseases-10 (ICD-10) [32] codes: F 32, 33, 34, 34, 38, 40, 41 (anxiety, depression or mixed affective states).

• Each participant will also require an informant, who has known them for at least six months, and who will also be available throughout the study to complete assessments.

*Exclusion criteria*:

• Participants with co-morbid conditions of substance misuse, autism and those currently receiving psychological treatment.

• Service users with moderate/severe ID.

### Randomisation

The randomization procedure (randomized permuted blocks) will be supervised by Robert Blizard, statistician, and administered by a departmental secretary (allocation concealment). The research assistant who will carry out the clinical and cost assessments (at baseline, end of treatment and follow-up) will remain blind to the treatment allocation of the participants until they have reached the end of the study.

### Intervention: Manualised Individual Cognitive Behavioural Therapy (CBT)

The participants will receive up to 16 weekly one-to-one treatment sessions of 60 minutes each offered over a period of 4 months. The CBT will be delivered by qualified CBT. The CBT manual will be developed in the first phase of the study by the research team comprising of specialists in the fields of intellectual disabilities, CBT and health interventions as well as clinical psychologists and speech and language therapists who have worked with people with intellectual disabilities.

A support worker also will be employed for the trial to help the participants through the treatment. His/her duties will involve facilitating appointments for those receiving CBT session and assist homework tasks.

The CBT therapists delivering the intervention will be supervised by the Consultant CBT Therapists (MS) and the support worker will be supervised by the Consultant Psychiatrist (AH).

### Control Group

Treatment As Usual (TAU) is defined as the standard treatment that would be available to any adult with an intellectual disability who has been referred to the intellectual disability service. This includes care management, medical, nursing, psychological input and/or social support.

### Primary outcomes

Beck *Anxiety and Depression Inventory Youth (BAI-Y & BDI-Y)*: Two subscales from the Beck Youth Inventories (BYI) [33] will be used to measure the severity of the cognitive accepts of depression and/or anxiety. This is a self-report assessment where each subscale consists of 21-items that are rated along a four- point Likert scale ('never', 'sometimes', 'often' or 'always'). The participant is required to rate each item in the scale that best represents their current mood especially during the last two weeks. Both subscales will be administered at baseline, end of treatment (four months) and at a six months follow-up. We have chosen the Youth Inventories because they are particularly sensitive to cognitive change and brief and simple in their language which will only require minimal change (for example the word "school" will be replaced by "college" or "work"). This point is of special importance in people with ID who may have difficulty in self reporting thoughts and emotional states. A questionnaire that is couched in accessible terms is invaluable in assisting people with mild ID to communicate their emotional and social distress. In addition, the service users will be offered assistance to clarify terms by the researcher if necessary. Furthermore, research has shown that the Beck Inventories can reliably and consistently be used with people with mild ID [34].

### Secondary Outcomes

*Client satisfaction questionnaire (CSQ-8) *[35]: Satisfaction with treatment will be measured by using a modified client satisfaction self-assessment questionnaire that consists of eight statements of satisfaction eliciting the client's perception of the mental health service/intervention rated on a four point Likert scale at 4 months from baseline (i.e. end of treatment).

*Manchester Short Assessment of Quality of Life (MANSA) *[36]: This is a quality of life questionnaire that consists of 16-items derived from the Lancashire Quality of Life Profile [37]. MANSA comprises 4 objective questions and 12 subjective questions. The subjective items assess satisfaction with life as a whole, employment status, financial situation, number and quality of friendships, leisure activities, accommodation, personal safety, living arrangement ( living with others or alone), sex life, relationship with family, physical health and mental health. Each item is rated on a seven-point satisfaction scale, from 1 = 'Couldn't be worse' to 7 = 'Couldn't be better'. This questionnaire will be administered at baseline and at 4 months (i.e. end of treatment).

*Client Service Receipt Inventory (CSRI) *[38]: this is a validated tool used to evaluate the cost and use of health resources and services by service users with psychiatric problems and learning disabilities [39,40]. The questionnaire will be tailored to suit the data requirements and broad approach to data collection for this trial. We will record information such as the use (frequency and duration) of health and social care services, hospital attendance and admissions, accommodation and living situations, educational services, income, employment and benefits. Data will be collected directly from the service user, support workers' and from the service records to determine service use. Costs will be measured at baseline and at 4 months (i.e. end of treatment).

#### Qualitative interviews

We will also carry out fifteen minutes interviews' consisting of open ended questions with prompts that will focus on obtaining the participant and their carer or support worker's experiences and the process of therapy. These will be administered once the intervention has been completed. The interviews will be analysed using NVivo software for themes, commonalities and differences in opinions.

### Treatment Manual

The final version of the manual will be informed by the practical use of the manual and the therapists' feedback. A professional writer will assist with producing a high quality document to allow for an accessible and engaging manual that can be utilised effectively.

### Treatment process evaluation

It has been suggested that there are three factors that are important for successful psychotherapeutic interventions: treatment delivery, receipt and enactment [41]. Treatment delivery aims to determine whether the proposed treatment is actually being given by the therapist. Modified CBT will be delivered by a therapist accredited or accreditable with the British Association for Behavioural and Cognitive Psychotherapies. All sessions will be audio taped. The therapist will keep a diary account of his/her interventions. Random samples of 1 in 10 therapy sessions (CBT) will be taken and rated using the Revised Cognitive Therapy Scale [42] to test its application in people with ID. A score of 39 or more will be taken as indicating adequacy of CBT treatment. Adherence to therapy will be measured using the checklist that has been devised in the CBT manual developed for the purpose of the study. The checklist will ensure that essential ingredients are covered by the therapist and if not, then the reason shall be recorded.

Treatment receipt is concerned with whether the treatment given is actually understood by the patient. In order to evaluate this, accessible materials (home/worksheets) will be developed as part of the manual. This process will be led by SM and an Accessible Information Worker who has been developing accessible materials for people with a range of intellectual disabilities for several years. The accessible home/worksheets will be discussed with a service users' consultation group with whom we have already established working relationships as a result of other service related projects.

Treatment enactment suggests that even if the treatment is delivered and understood, it will not be effective unless the patients act on this. To determine this aspect the feasibility of the homework tasks will be checked by piloting a selection of interventions with service users and determining whether they can be undertaken and if not why not. We anticipate that individuals will have difficulties with motivation or ability to complete their homework, therefore, have enlisted the assistance of the study support worker in encouraging them to do so.

### Statistical analysis

The data will be managed using SPSS (v 14). The analyses will focus principally on descriptive data on recruitment rates, characteristics of participants, attrition from therapy and research and the function of the study manual. We will describe the techniques employed and documented by the therapists. The primary outcomes (mean scores and confidence intervals) will be compared at baseline, four months and six months post randomisation for both trial arms. We shall determine the variance of our outcome measures and the effect size in this feasibility trial in order to inform the power calculation for a definitive RCT. We shall also conduct exploratory analyses of factors predicting treatment outcome such as receipt of medication (antidepressant), age, course of illness, treatment preference and number of sessions received.

All analyses will be by intention-to-treat. The study will assess the feasibility of recruitment and attrition from the intervention; applicability of the manualised CBT and participants' and informants' views of the treatment. We will collect demographic data on all those approached; will determine the proportion of people suitable and those who engage in the study. We will calculate the means and standard deviations for the BDI-Y and the BAI-Y. Although caution is necessary as the variance of the outcome scores in a feasibility study, it may not truly reflect this in a future trial. The data will be explored to identify possible predictors of outcome and factors related to engagement and or attrition.

A descriptive analysis of total costs and individual resource use components (e.g. primary care, therapy) will be conducted.

Whilst the proposed sample size (*n *= 30) for this trial is too small to test for statistically significant differences in clinical or social outcomes between intervention and control groups, it will investigate the feasibility of the intervention and likely effect size and recruitment potential.

### Ethical considerations

This trial has been approved by the Joint UCL/UCLH Committee on the Ethics of Human Research, Committee Alpha (reference number: 08AL 332).

#### Informed consent and information sheets

The information sheets and consent forms were developed in an accessible (i.e. easy-to-read) format by an accessible information worker as before. The information sheets contained a succinct standard script written in accessible language to be used as an aid to verbally inform the participants about the trial before written informed consent is sought from the individuals who are willing to enter the study. It is essential when working with people with intellectual disabilities to ensure that information is understood by frequently checking and asking the individuals to repeat the information in their own words. Information should also be concise to avoid information overload which can be counterproductive. The participants also will be given information sheets to take away and go over with their preferred carer or support worker in their own time and pace.

#### Withdrawal from the trial

Participants may voluntarily withdraw from the trial for any reason. The withdrawal of the patient from the trial will not affect their access to treatment by the relevant intellectual disability service.

#### Confidentiality

Each participant will be assigned a unique trial number in order to store and identify their trial data. All data for the duration of the study will be kept in locked cabinets in the researcher's office and on a password locked computer. The data protection policy of the University College Medical School will be observed for this project, which states a 10 year data storage policy.

## Discussion

This will be the first randomised controlled trial to evaluate a manualised individual cognitive behavioural therapy in treating common affective disorders in people with mild intellectual disabilities. Successful completion of the pilot randomised controlled trial will show it is feasible to recruit participant with intellectual disabilities to a RCT and to deliver the treatment. The results from this study are likely to have considerable policy impact regarding accessibility of psychological treatments by people with intellectual disabilities in line with the government's policy for Improving Access Psychological Therapies (IAPT). Also, it will add to the evidence base for interventions in people with intellectual disabilities, which is currently sparse [43].

Demonstrating clinical and cost effectiveness of modified CBT along with the identifying the clinically useful elements of the treatment manual would have significant benefit in relation to current policies in the UK set out by the National Institute of Clinical Excellence [44] which advocates CBT as a preferred and effective form of treatment for depression and anxiety disorders in the general population and Valuing People [45] which states that people with intellectual disabilities should have the same access to healthcare as people without intellectual disabilities.

## Abbreviations

BAI-Y: Beck Anxiety Inventory Youth; BDI-Y: Beck Depression Inventory Youth; CBT: Cognitive Behavioural Therapy; CSQ: Client satisfaction questionnaire; CSRI: Client Service Receipt Inventory; ICD-10: International Classification of Diseases - 10; ID: Intellectual Disabilities; MANSA: Manchester Short Assessment of Quality of Life; PAS-ADD: Psychiatric Assessment Schedules for Adults with Developmental Disabilities; RCT: Randomised Controlled Trial; SPSS: Statistical Package for the Social Sciences; TAU: Treatment as usual.

## Competing interests

The authors declare that they have no competing interests.

## Authors' contributions

AH, MK and MS conceived and wrote the funding application. AS, MS, MK, AS, SM and CP contributed towards the developing the therapists CBT manual. SM and KA were involved in the development of the accessible information. KA will be acquiring the data and maintain the dataset. RB and KA will be involved in the data handling and analysis whilst all authors will be involved in the data interpretation and dissemination.

All authors read and approved the final manuscript.
